# Sealing ability and microbial leakage of root-end filling materials: MTA versus epoxy resin: A systematic review and meta-analysis

**DOI:** 10.1016/j.heliyon.2021.e07494

**Published:** 2021-07-07

**Authors:** Mario Dioguardi, Mario Alovisi, Diego Sovereto, Giuseppe Troiano, Giancarlo Malagnino, Michele Di Cosola, Angela Pia Cazzolla, Luigi Laino, Lorenzo Lo Muzio

**Affiliations:** a Department of Clinical and Experimental Medicine, University of Foggia, Via Rovelli 50, 71122, Foggia, Italy; b Department of Surgical Sciences, Dental School, University of Turin, Turin, Italy; c Multidisciplinary Department of Medical-Surgical and Odontostomatological Specialties, University of Campania “Luigi Vanvitelli”, 80121, Naples, Italy

**Keywords:** Sealing ability, Microbial leakage, Endodontic, Epoxy resin, MTA

## Abstract

**Objectives:**

The purpose of this systematic review of the literature is to investigate which of the epoxy-based cements and those based on Tricalciumsilicate (MTA, Bioceramic) have the best sealing capacity through the analysis of studies that have provided a survey model in vitro of bacteria leakage.

**Source:**

The articles were identified using electronic databases such as PubMed, Scopus, the search was conducted between 8.12.2020 and 31.12.2020 and a last search was conducted on 2.12.2021.

**Study selection:**

678 records were identified and after removing the duplicates we obtain 481 records, with the first phase of screening and selection of records we reached 204 and with the application of the inclusion and exclusion criteria we selected 31 articles, only 9 studies made a direct comparison between the two endodontic cement categories and presented data that could be included in the metaanalysis.

**Data:**

The meta-analysis of first outcome shows an odds ratio of 2.70 C.I.(Confidence Interval) [1.54, 4.73], the test for overall effect has a p value = 0.0005 with a heterogeneity index of I 2 of 9%; The second outcome meta-analysis shows an Odds Ratio of 1.50 C.I. (Confidence Interval) [0.92, 2.46] with a p value of 0.10 with an I 2 of 79%.

**Conclusion:**

the sealing ability is higher for epoxy resins than for tricalcium silicate-based cements, for observation periods longer than 90 days.

**Clinical relevance:**

The knowledge of the cement that determines the best sealing ability and resistance to microbial leakage, can be of help for the dentist who has to face clinical situations such as endodontic retreatments whose failure is determined by the persistence of bacteria in the endodontic canals.

## 1 Introduction

One of the fundamental objectives of endodontic treatment is to establish a seal, which is as durable and predictable as possible over time, surrounding the apical region at the root of a tooth, ensuring the coronal seal is guaranteed by definitive restorations or crowns. Cements and resin or gutta percha cones are used to establish an apical seal, and it is essential that these materials have excellent sealing ability and resistance to microbial leakage [1, 2]. According to Komabayashi et al. (2020), the cementitious materials used in endodontics can be classified as follows: zinc oxide-eugenol, salicylate and tricalcium silicate (MTA and bioceramics), zinc oxide-fatty acid, glass ionomer, silicone, epoxy resin, and methacrylate resin. Recently, tricalcium silicate-based cements (MTA and bioceramics) have received a lot of attention for their high biocompatibility with osteo-inductive regenerative properties [3].

In addition to biocompatibility, effective sealing ability is fundamental for endodontic cements. The sealing ability of endodontic cements has been investigated using various methods including fluid infiltration [4, 5], radioisotope [6, 7], dye penetration [8], and loss of bacteria [8, 9]. Among the most commonly used methods, and the method clinically closest to the cause of endodontic failure, is a model based on the release of microorganisms such as *Enterococcus faecalis*, which is one of the microorganisms involved in secondary endodontic infections [10].

Recent studies on the sealing ability of tricalcium silicate-based cements (MTA and bioceramics) have shown non-superior performance compared to other endodontic cements. For example, Yanpiset et al. (2018) reported no statistically significant difference in bacterial leakage between a bioceramic sealant and epoxy resin, while Jafari et al. (2016) published an in vitro study on sealing ability and concluded that epoxy resin showed the lowest bacterial leakage as compared with MTA [11, 12]. Among the studies in the literature, there are some discrepancies in the results on the sealing ability of different cements. Since the epoxy- and tricalcium silicate-based cements (MTA and bioceramics) are different in their composition and biological properties, we decided to ask the following review question: Which of these two endodontic cements, epoxy- or tricalcium silicate-based cements, had the best sealing ability in an in vitro model of bacterial leakage?

The compositions of the two main epoxy resin-based cements, as reported by Komabayashi et al. (2020) are as follows: for AH-26 (bismuth oxide, hexamethyleneteramine, silver powder, titanium oxide, and bisphenol A diglycidyl ether) and for AH Plus (bisphenol A-based epoxy resin, zirconium oxide, bisphenol F based-epoxy resin, calcium tungstate, iron oxide, silica, N, N-dibenzyl-5-oxanonadiamin-1,9, amantiameamine, tricyclodecane-diamine, calcium tungstate, and zirconium oxide) [3].

The direct antimicrobial effects for epoxy resin-based cements, seem to be slightly lower as compared with those based on zinc oxide-eugenol [13]. In addition, higher cytotoxicity has been found towards fibroblasts as compared with other types of cements, along with a genotoxic effect for AH-26 due to the release of formaldehyde, which has not been found for AH Plus [14]; however, biocompatibility is higher than in zinc oxide-eugenol-based cements [15].

The composition of the main tricalcium silicate-based cements (MTA and bioceramics) are as follows: MTA Fillapex (Angelus, Brazil): methyl salicylate, butylene glycol, colophony, bismus trioxide, fumed silica, titanium dioxide, Paste B fumed silica, titanium dioxide, tricalcium silicate, dicalcium silicate, calcium oxide, tricalcium alminate, pentaerythritol rosinate, and p-toluenesulfide; Total Fill BC: zirconium oxide, calcium silicates, calcium phosphate, calcium hydroxide, filler, thickening agents; BioRoot RCS: tricalcium silicate, zirconium oxide, and aqueous solution of calcium chloride. Some authors do not consider MTA Fillapex to be a tricalcium silicate because its composition contains resin; in fact, Komabayashi places it among the silicates [3], in contrast to many other authors [16, 17].

Studies on the antimicrobial effects of tricalcium silicate-based cements (MTA and bioceramics) have reported differing results. Torabinejad et al. reported an antimicrobial effect on facultative bacteria but not on facultative anaerobes. Tanomaru-Filho et al. (2007) reported antimicrobial activity similar to other cements [18], while Estrela did not report any antimicrobial activity directed towards *E. faecalis*, *S. aureus*, *C. albicans*, and *B. subtilis* [19]. The antimicrobial effect seemed to depend on an increase in pH and the release of calcium hydroxide ions [20]. Most of the studies are in agreement in establishing an excellent biocompatibility superior to many other classes of tricalcium silicate-based cements with osteo-inductive properties [21].

Our hypothesis is that between the two types of cements (epoxy- and tricalcium silicate-based), there are differences in the ability to seal the apical region of a tooth and in the resistance to bacterial infiltration.

## 2 Materials and methods

This study was conducted with reference to the guidelines described by the preferred reporting items for systematic review and meta-analysis (PRISMA) [22].

The following PICO framework was followed: participants—root apexes of extracted teeth sealed with endodontic cements; intervention—sealing of the apical third in an in vitro model with bacterial infiltration; comparison—closure of the apical third of the tooth with 2 different types of endodontic cements (tricalcium silicate- and epoxy resin-based); outcome—sealing ability of cements measured through the odds ratio of infiltrated and non-infiltrated samples between the 2 types of endodontic cements (tricalcium silicate- and epoxy resin-based cements).

In this study, we aimed to answer the following PICO question: Which of the 2 types of endodontic cements, i.e., tricalcium silicate- or epoxy resin-based, has the best sealing ability in an in vitro model with bacterial infiltration, based on calculating the odds ratio between infiltrated and non-infiltrated samples, during the meta-analysis of the extracted data?

On the basis of the title and the abstract of numerous scientific studies, all in vitro and ex vivo studies on the sealing ability of endodontic cements based on a bacterial leakage model were considered to be potentially admissible.

The exclusion criteria applied to the studies were the following: not written in English, with infiltration measurement for a period less than 60 days, not ex vivo or in vitro models, and data not provided on the number of infiltrated samples at the end of the experimental observation period. Clinical cases, clinical trials, reviews (the reviews were considered as sources of bibliographic information, studied, and analyzed in a preliminary phase so as not to repeat a systematic review already performed by previous authors), were excluded from this systematic review.

The inclusion criteria applied to the studies were as follows: All in vitro and ex vivo studies that report data on the number of infiltrated samples for a period of at least 60 days for both types of endodontic cement and which present a low risk of bias. The inclusion and exclusion criteria are summarized in Table 1. The articles deemed suitable were read and analyzed in order to include them in the qualitative and quantitative analyses.

**Table 1:** Inclusion and exclusion criteria.

Category	Exclusion Criteria	Inclusion Criteria
Publication Language	Not English	English
Study types	Review, Systematic review, case report, case series, Clinical Study, study in vitro not ex vivo.	Study in vitro ex vivo^1^, performed on a bacterial leakage study model.
data characteristics	Report data over a period of less than 60 days.	Report data on the number of infiltrated samples in a period of over 60 days, report data on both types of cement (Tricalciumsilicate and epoxy resins).
Risk of Bias	High risk of bias.	Medium o low risk of bias.

1 Performed on extracted teeth.

### 2.1 Research methodology

The articles were identified using electronic databases such as PubMed and Scopus. The search was conducted between 8 and 31 December 2020 and the final search was conducted on 1 February 2021.

All keywords used and the related database search details are explicitly shown in Table 2.

**Table 2:** Overview of the search methodology; Records identified by databases:678, 485 after removing overlaps. Articles included in meta-analysis: 9.

Database - Provider	Key words	Search Details	Number of records	articles After removing overlaps articles	remaining articles that dealt with the issue of sealing ability for endodontic cements under review	Article remaining after applying the inclusion and exclusion criteria	Articles included in meta-analysis
Pub med	"epoxy resin sealer"	"epoxy resin sealer"[All Fields]	53				
Pub med	calcium silicate sealer	("calcium silicate"[Supplementary Concept] OR "calcium silicate"[All Fields]) AND ("sealer"[All Fields] OR "sealers"[All Fields]) Translations calcium silicate: "calcium silicate"[Supplementary Concept] OR "calcium silicate"[All Fields] sealer: "sealer"[All Fields] OR "sealers"[All Fields]	181				
Scopus	epoxy resin sealer"	TITLE-ABS-KEY (“epoxy resin sealer”)	77				
Scopus	calcium silicate sealer	TITLE-ABS-KEY (“calcium silicate sealer”)	17				
Pub med	bioceramic AND endodontic	("bioceramic"[All Fields] OR "bioceramics"[All Fields]) AND ("endodontal"[All Fields] OR "endodontic"[All Fields] OR "endodontical"[All Fields] OR "endodontically"[All Fields] OR "endodontics"[MeSH Terms] OR "endodontics"[All Fields]) Translations bioceramic: "bioceramic"[All Fields] OR "bioceramics"[All Fields] endodontic: "endodontal"[All Fields] OR "endodontic"[All Fields] OR "endodontical"[All Fields] OR "endodontically"[All Fields] OR "endodontics"[MeSH Terms] OR "endodontics"[All Fields]	226				
Scopus	bioceramic AND endodontic	TITLE-ABS-KEY (bioceramic AND endodontic)	124				
Web of science	epoxy resin sealer	You searched for: TOPIC: (epoxy resin sealer) Timespan: All years. Indexes: SCI-EXPANDED, SSCI, A&HCI, CPCI-S, CPCI-SSH, BKCI-S, BKCI-SSH, ESCI, CCR-EXPANDED, IC.	337				
Web of science		You searched for: TOPIC: (bioceramic AND endodontic) Timespan: All years. Indexes: SCI-EXPANDED, SSCI, A&HCI, CPCI-S, CPCI-SSH, BKCI-S, BKCI-SSH, ESCI, CCR-EXPANDED, IC.	115				
			1130	481	204	31	9

The research methodology was carried out in 4 phases.

In the first phase, the method for identifying the records was chosen taking into consideration the following points:

a. Choice of 2 reviewers with the task of identifying records and a 3rd reviewer with the task of resolving doubtful situations;
b. Choice of databases and providers;
c. Choice of keywords;
d. Decisions on inclusion and exclusion criteria.

The second phase involved the identification of the records on the databases (the duplications were removed through the use of EndNote 9 software), the screening of potentially eligible articles (through an analysis of the title and abstract), and the choice of articles to be included in the meta-analysis.

The third phase involved the comparison of the studies identified by the 2 independent reviewers and the choice of articles to be included in the meta-analysis (the k-agreement between the 2 reviewers was 0.84). During this phase, it was decided to also perform an additional meta-analysis data analysis which included an observation period of the tested samples of 90 days.

The fourth phase involved the extraction of data by the 2 reviewers independently with subsequent comparison of the extracted data.

The data sought in the studies by the two reviewers concerned the total number of samples with microbial leaking for the 2 types of endodontic cements (one type based on tricalcium silicate and the other type based on epoxy resins).

### 2.2 Statistical analysis protocol

The protocol for the meta-analysis was conducted based on the guidelines from the *Cochrane Handbook for Systematic Reviews of Interventions*. The program used to perform the meta-analysis was Reviewer Manager 5.3 (Cochrane Collaboration, Copenhagen, Denmark). The odds ratio between the two types of endodontic cements was measured taking into consideration the number of total samples and the number of samples with microbial leakage for each study included in the meta-analysis. The presence of heterogeneity was measured with the Higgins index (*I*^2^); values above 50% were considered to be heterogeneous. The risk of bias within the studies was assessed following the PRISMA guidelines for assessing the quality of studies in meta-analyses. The meta-analysis results were graphically depicted using a forest plot and the heterogeneity results using a funnel plot.

## 3 Results

From the searches in the PubMed, Scopus, and Web of Science databases, 1130 articles were initially identified. EndNote software was used to remove duplicates with 481 articles remaining. After the first phase of screening and selection of records, we identified 204 articles, and after the application of the inclusion and exclusion criteria, we selected 31 articles. There were only nine studies that made a direct comparison between the two types of endodontic cements and presented data that could be included in the meta-analysis.

We included the following nine articles in the meta-analysis:

• six articles for Outcome 1, i.e., all studies reporting data on a number of infiltrated samples for a period of at least 90 days;
• seven articles for Outcome 2, i.e., all studies reporting data on a number of infiltrated samples for a period of at least 60 days.

**Figure 1:**
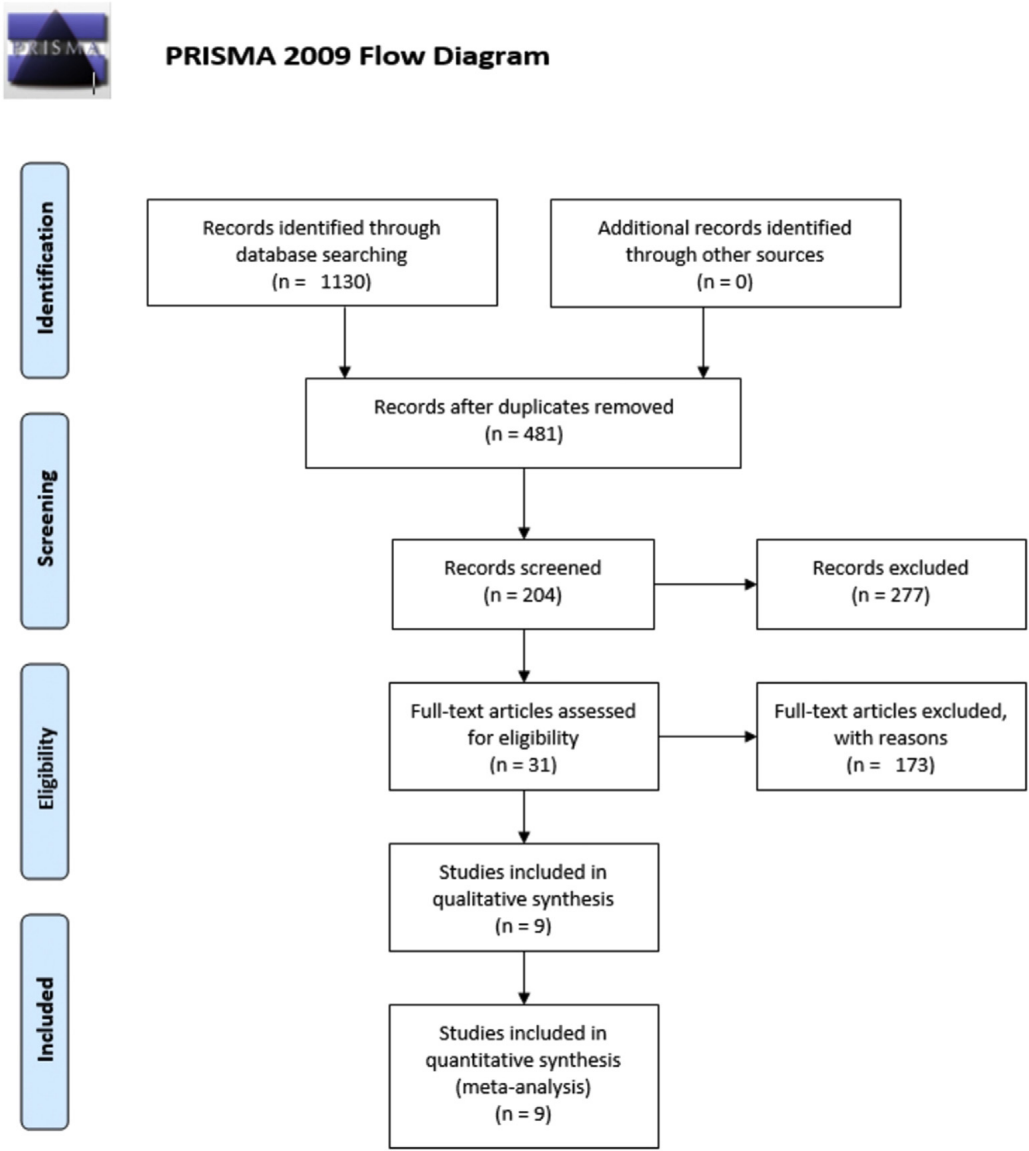
PRISMA 2009 flow diagram.

All selection and screening procedures are described in the flowchart shown in Figure 1.

### 3.1 Study characteristics and data extraction

The included studies for the quantitative analysis were: For Outcome 1, Jafari (2016) [12], Medeiros (2016) [23], Milani (2019) [24], Oliveira (2011) [25], Razavian (2016) [26], and Reyhani (2015) [27]; for Outcome 2, Yanpiset (2018) [11], Amezcua (2015) [28], Medeiros (2016) [23], Oliveira (2011) [25], Razavian (2016) [26], Reyhani (2015) [27], and Antunovic (2021) [29].

The extraction of the data and the methods in which they have been reported follow the indications of the *Cochrane Handbook for Systematic Reviews of Interventions*.

The extracted data included the journal information (first author, data, and reference); the type of endodontic cement investigated, the number of samples examined, the number of samples showing bacterial infiltration with the elapsed time period, and the bacterial species used. If the data were reported as a percentage, the number was calculated through the use of proportions (Table 3).

**Table 3:** Number of total and leaked samples, based on the time elapsed (10–120 days). MTA-PG: Mineral trioxide aggregate, Propylene glycole; AH26 (Dentsply, DeTrey, Konstanz, Germany) and MTA Fillapex (Angelus, Londrina, Brazil), Apatite Root Canal Sealer (Sankin-Kogyo, Tokyo, Japan), CPM: Portland cement (EGEO SLR, MTM Argentina SA, Buenos Aires, Argentina), MTA (Angelus, Londrina, Paraná, Brazil), MBPc (University of São Paulo, Brazil, epoxy resin sealer containing calcium hydroxide), AH Plus (Dentsply DeTrey, Konstanz, Germany), Resilon (Real Seal®, Sybron Endo, Glendora, USA), Super-EBA (Harry J. Bosworth, Skokie, IL, USA), ProRoot MTA (Dentsply Sirona), AHP: AH Plus, ESE: Epiphany SE (Pentron Clinical Technologies, LLC., Wallingford, CT, USA); SEL: Sealapex (Kerr Corp., CA, USA); AGP: Activ GP (Brasseler USA, Dental Instrumentation, USA); EDF: Endofill, (Dentsply Industria e Comercio Ltda, Petropolis, RJ, Brazil); CPM: Endo CPM Sealer (EGEO S.R.L. under license of MTM Argentina S.A., Buenos Aires, Argentina); MTAS: MTA Sealer (Araraquara Dental School, UNESP, Brazil); BCS: bioceramic sealer (Totalfill BC Sealer, FKG Dentaire SA, La Chaux-de-Fonds, Switzerland); GP: gutta percha; BCC: bioceramic-impregnated gutta percha cone; BioRoot RCS: BioRoot root canal sealer (Septodont, Saint Maur-des-Fosses, France).

First author, data, reference	endodontic cements tested	number of samples		number of samples with leakage										Bacteria
				10	20	30	40	50	60	70	80	90	120 days	
Milani, 2019 [4]	MTA-PG in dry canals	15	45									7		*Enterococcus faecalis*
	MTA-PG in wet canals	15										12		
	MTA fillapex	15										14		
	AH26	15	15									10		
Jafari 2016 [2]	AH26	44	44									7		*Enterococcus faecalis*
	MTA fillapex	44	44									10		
	Apatite Root Canal Sealer	44										11		
Medeiros 2016 [3]	white MTA,	20				7			7			7	7	*Enterococcus faecalis*
	CPM,	20				4			4			4	4	
	MBPc	20				1			1			2	2	
Amezcua 2015 [9]	SuperEBA	10												*Staphylococcus aureus Enterococcus faecalis,* *Pseudomonas aeruginosa, Bacillus subtilis, Candida albicans.*
	RealSeal® thermoplasticized	10							9					
	ProRoot® MTA	10							10					
	Thermoplasticized gutta-percha + AH Plus®	10							7					
Oliveira 2011 [5]	AHP	15	30			5			7			8	9	*Enterococcus faecalis*
	S26	15				8			9			9	10	
	ESE	15				4			9			11	12	
	SEL	15				7			7			8	11	
	AGP	15				6			11			14	14	
	EDF	14				7			7			11	12	
	CPM	15				9			10			12	13	
	MTAS	13	13			8			10			11	12	
Razavian 2016 [6]	AH 26	25							5					*Enterococcus faecalis*
	MTA Fillapex	25							16					
Reyhani 2015 [7]	AH Plus	15		11	12	14	14	14	14	14	15	15		*Enterococcus faecalis*
	AH Plus post	15		11	12	14	14	14	14	15	15	15		
	MTA Fillapex	15		7	8	8	9	9	11	13	14	15		
	MTA Fillapex post	15		6	8	9	10	10	11	14	15	15		
Yanpiset 2018 [8]	GP/AH plus	20							4					*Enterococcus faecalis*
	BCC/AH plus	20							9					
	GP/BCS	20							9					
	BCC/BCS	20							5					
Antunovic 2021 [10]	BCS	14	56			0			2					*Enterococcus faecalis*
	BioRoot RCS	14				1			5					
	MTA Fillapex	14				2			5					
	MTA Plus	14				5			7					
	AH Plus	14	14			1			11					

### 3.2 Risk of bias

The risk of bias was assessed based on the Checklist for Reporting In vitro Studies (CRIS) guidelines [30] proposed to evaluate in vitro dental studies. The results are shown in Table 4; each type of cement was assigned a value from one to five (where one = low quality and five = high quality). The questions that the reviewers answered by assigning a score were the following:

1. For the sample size calculation, “Is the sample size adequate for obtaining statistically significant results?”
2. For meaningful difference between groups, “Has the ‘meaningful difference’ measurement been set correctly in the groups taking into account the sample size and the type of measurement?”
3. For sample preparation and handling, “Does the study describe information on the production or handling of the samples to be tested?”
4. For allocation sequence, randomization, and blinding, “Did the samples have equal and independent possibility of a sample entering any group?”
5. For statistical analysis, “Are the statistical methods described?”

**Table 4:** Assessment of the risk of bias within the studies, with scores 7 to 12 = low quality, 13 to 20 = intermediate quality, and 21 to 25 = high quality.

First Author, Data	Sample size calculation	Meaningful difference between groups	Sample preparation and handling	Allocation sequence, randomization and blinding	Statistical analysis	Score	outcome
Milani, 2019 [4]	3	3	4	3	4	17	1
Jafari 2016 [2]	4	3	3	3	4	17	1
Medeiros 2016 [3]	4	3	4	3	3	17	1, 2
Amezcua 2015 [9]	2	3	4	4	4	17	2
Oliveira 2011 [5]	3	3	3	2	3	14	1, 2
Razavian 2016 [6]	4	3	4	4	2	17	1, 2
Reyhani 2015 [7]	3	3	4	3	3	16	1,2
Yanpiset 2018 [8]	4	3	4	3	4	18	2
Antunovic 2021 [10]	3	3	4	3	4	17	2

Studies presenting a high risk of bias were not included in the meta-analysis and were eliminated during the inclusion phase (Table 4). The assessment of the risk of bias of the nine included articles was conducted by M.D.

**Figure 2:**
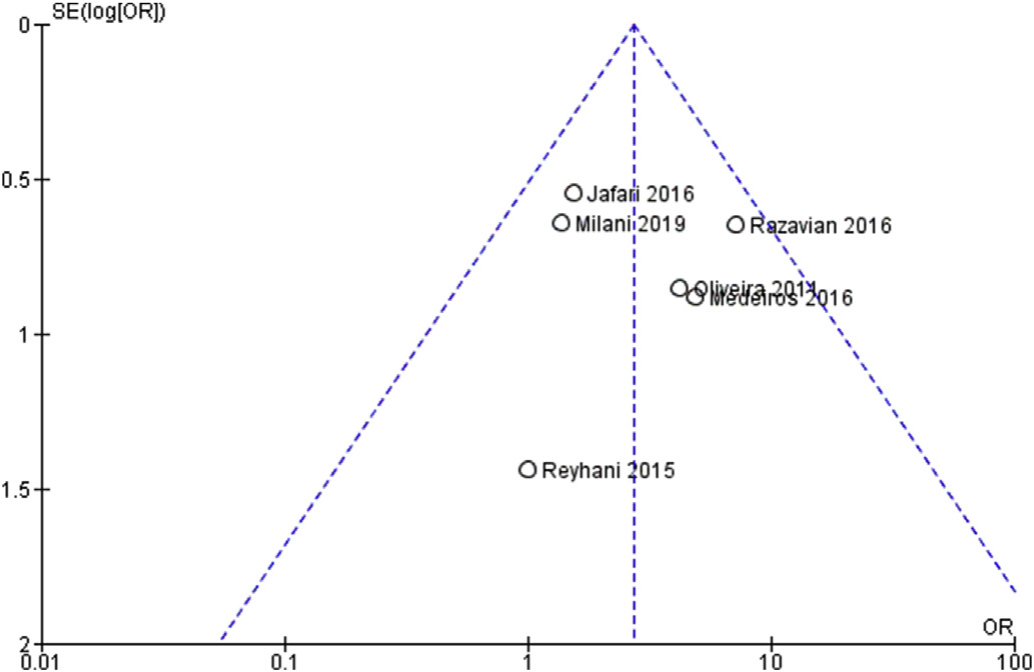
Funnel plots of the evaluation of heterogeneity for first outcome.

The risk of bias among the studies for Outcome 1 is considered to be low; the heterogeneity showed a value represented by *I*^2^ (Higgins's index) of 9% (heterogeneity values greater than 50% are considered to be high) with a *p*-value < 0.36 from the Chi-squared test. The high heterogeneity is also confirmed by the funnel plot in Figure 2.

**Figure 3:**
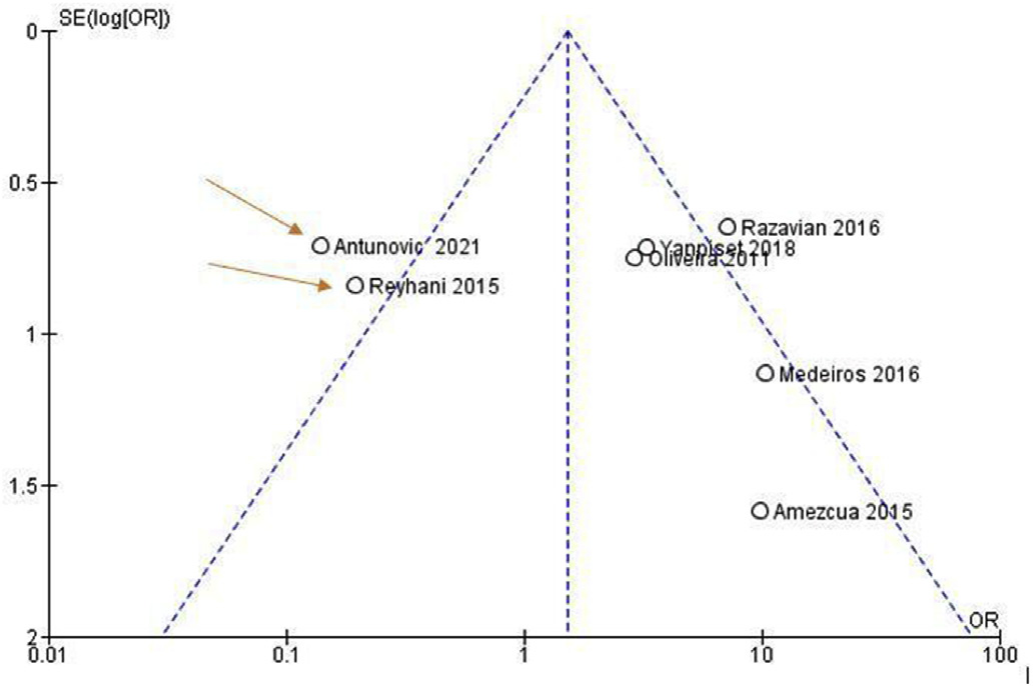
Funnel plot for the secondary outcome. The arrows highlight the sources of heterogeneity (*I*^2^ 79%) Antunovic 2021, Reyhani 2015.

The risk of bias among the studies for Outcome 2 is medium; the heterogeneity between the studies is considered to be high and the *I*^2^ index stands at a value of 79%b. A visual analysis of the funnel plot shows a study as a probable source of heterogeneity and bias between the studies (Reyhani (2015) [27] and Antunovic (2021) [29]) Figure 3. Therefore, we decided to perform a sensitivity analysis with an assessment of the confidence intervals of the individual studies to confirm the sources of heterogeneity.

### 3.3 Meta-analysis

Statistical data analysis was performed using Rev Manager 5.4 (Cochrane Collaboration, Copenhagen, Denmark). The results were represented by forest plots.

**Figure 4:**
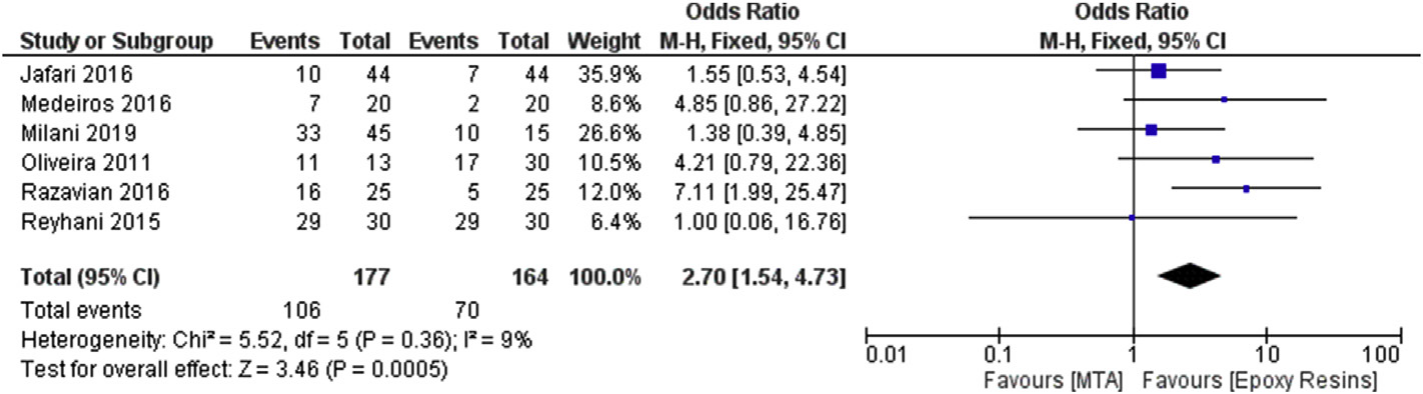
Forest plot of the fixed effects model of the meta-analysis of the first outcome.

The meta-analysis for Outcome 1 was conducted by applying fixed effects models given the low rate of heterogeneity (*I*^2^ = 9%). The meta-analysis shows an odds ratio of 2.70, with a confidence interval (CI) (1.54, 4.73). The test for the overall effect has a *p*-value = 0.0005 with an *I*^2^ of 9%. The forest plot presents a diamond positioned in favor of epoxy resin-based cements with a lower probability ratio of leakage for the tested samples after 90 days (Outcome 1) with 70 samples showing leakage as compared with a total of 164 samples (Figure 4).

**Figure 5:**
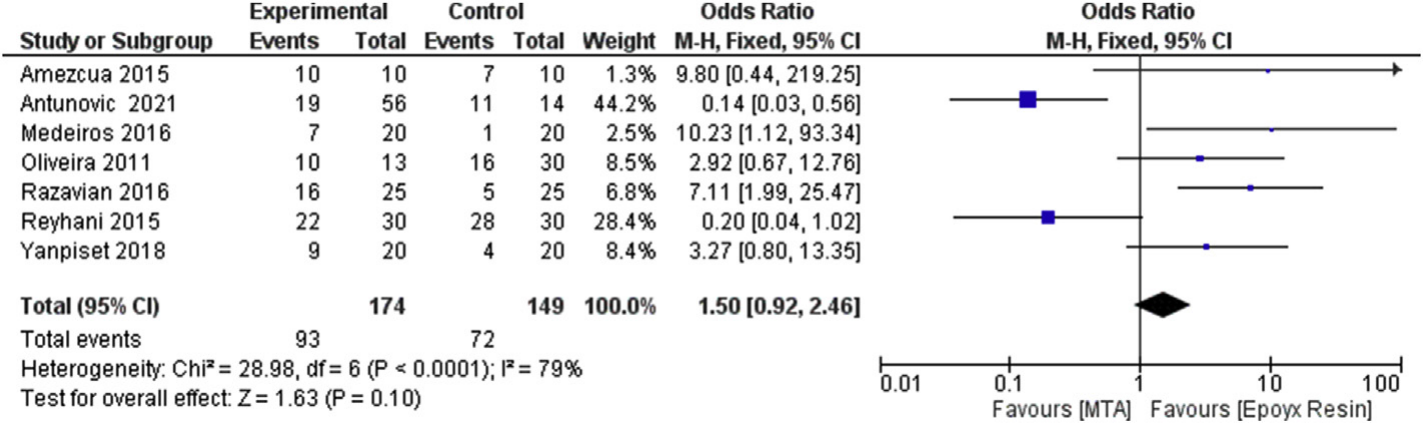
Forest plot of the fixed effects model of the meta-analysis of secondary outcome.

The meta-analysis for Outcome 2 shows an odds ratio of 1.50, confidence interval (CI) (0.92, 2.46), a *p*-value of 0.10, with an *I*^2^ of 79%. The forest plot is in favor of epoxy resin-based cements as compared with the MTA group but not in a statistically significant way, in fact, the central rhombus intersects the line of no effect (Figure 5). As a result that the heterogeneity was high, we decided to perform a sensitivity analysis in search of heterogeneity sources. From the visual analysis of the confidence intervals of the forest plot, it emerges that there is a poor overlap of the confidence intervals for the Reyhani (2015) [27] and Antunovic (2021) [29] studies (confirmed by the funnel plot).

**Figure 6:**
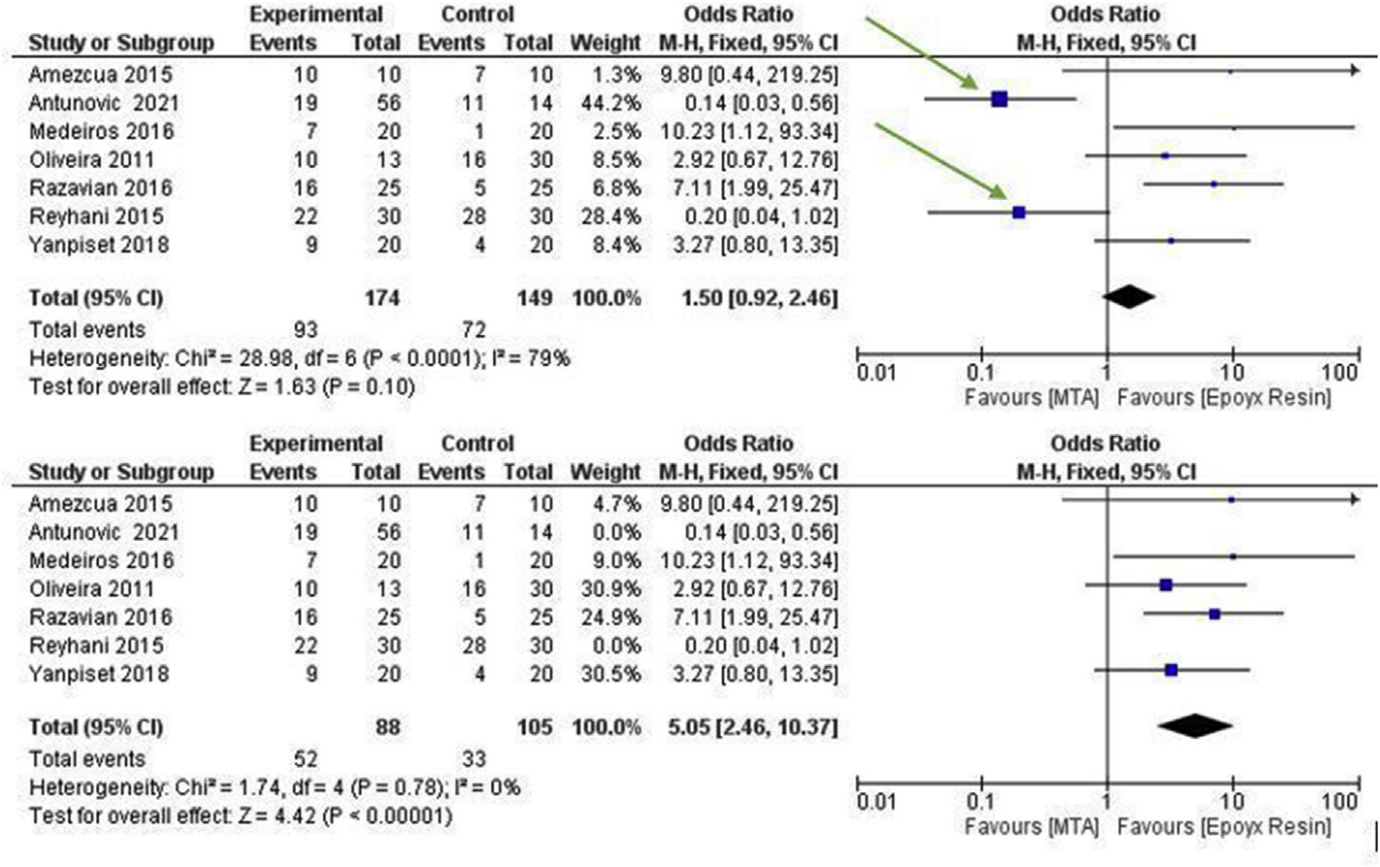
Sensitivity analysis: Forest plot of the meta-analysis of the secondary outcome; the arrows indicate the sources of heterogeneity that are identified by the funnel plot and are also evident on the forest plot.

By selectively removing the Reyahani (2015) and Antunovic (2021) studies, the heterogeneity (*I*^2^) goes from 79% to 0% and the forest plot still remains in favor of epoxy resin-based cements in a statistically significant way with an odds ratio of 5.05, CI(2 .46, 10.37), and *p*-value < 0.00001 (Figure 6).

## 4 Discussion

In the field of endodontics, one of the fundamental objectives for success is the achievement of an apical seal after a root canal, which must be guaranteed by the good seal of the material used.

All the studies that included a bacterial infiltration model involved the use of *Enterococcus faecalis* with the exception of Amezcua et al. [28], who also used other bacterial species.

The leakage model, in all included studies, involved the use of roots of mono-root elements sealed with endodontic cements, with the apex immersed from 2 to 4 mm in 5–10 mL of brain heart infusion (BHI), sterilized, sealed, and incubated at 37 °C from 24 h up to 3 days. Subsequently, *Enterococcus faecalis* was inserted and if there was leakage it was visualized through the variation of clarity of the BHI. The use of *Enterococcus faecalis* represents a proven and standardized model in several studies; furthermore, this bacterium has the ability to withstand concentrations of sodium hypochlorite at concentrations higher than 1% and is found mainly in persistent endodontic lesions [31, 32].

Razavian et al., among the studies included in the meta-analysis for both outcomes, reported statistically significant data in favor of epoxy resins; in fact, in the forest plot, the line representing the confidence intervals does not intersect the no effect line. Razavian concluded that AH-26 has a greater sealing ability as compared with MTA Fillapex. In agreement with the data of this study [26], Medeiros et al. [23] also reported statistically significant data in favor of epoxy resins (MBPc) as compared with MTA. Data in partial agreement with these studies but with no statistically significant results as reported by the forest plots of the two outcomes are the studies by Oliveira et al. (Outcomes 1 and 2) [25], Milani et al. and Jafary et al. (Outcome 1) [24], and Amezcua et al. and Yanpiset et al. (Outcomes 1 and 2) [11,28].

Oliviera et al. compared the 2 types of cements and also cements based on methacrylates and zinc oxide-eugenol, for a period up to 120 days, and concluded that the cements with the best sealing ability were AH Plus and Sealapex, while MTA was the cement that presented the worst sealing ability [25]. Data in partial contrast come from Reyhani et al. [27] who reported for both classes of cement an equal resistance to leakage.

Data from a 90-day sample observation period, from the studies included in the meta-analysis, report an odds ratio of 2.70 and CI [1.54, 4.73] with 70 samples showing leakage out of 164 samples for resin epoxy-based dements and 106 samples out of 177 samples for the MTA group.

These data are in line with Outcome 2, which measured the odds ratio of leaked and non-leaked samples of MTA and epoxy resins over an observation period of only 60 days, a shorter time period than Outcome 1, (odds ratio, 1.50; CI [0.92, 2.46]) with 72 leaked samples out of 149 samples for the epoxy resin-based cements and 93 leaked samples out of 174 samples for the MTA group.

Studies conducted on other models for testing the sealing ability have reported data in partial agreement with our review. Meidyawati et al. conducted a penetration study using inks on extracted teeth and compared mineral trioxide aggregate and resin epoxy sealer. They concluded that the sealing ability of MTA was lower than RES [33].

Microleakage studies using fluid infiltration models have presented data with non-statistically significant differences between epoxide resins and calcium silicate-based cements (MTA group) [34], in agreement with the data from the forest plot for Outcome 2.

Shourgashti (2018) [35] measured microleakage using a fluid transport model described by Wu et al. [36] and reported that the sealing ability of HealApex was comparable to that of AH-26, while, in the long term, the sealing of HealApex was based on epoxy sealant. Conclusions also in agreement with Amoroso-Silva et al., who compared resin-based cements (MBPc and S26) and calcium silicate-based cements (MTA and Portland cement), concluded that calcium silicate-based cements showed similar fluid filtration [34].

Asawaworarit et al. (2016) [37] reported that MTA Fillapex® had significantly more leakage than AH Plus® at 7 days, but at 4 weeks, MTA Fillapex® showed significantly better sealing ability than AH Plus® (*p* < 0.05). A study conducted by Ersahan et al. (2013) reported no difference between AH Plus and iRoot SP in terms of apical sealing capacity [38].

In addition, Gandolfi et al. (2010), in a study of sealing ability through the fluid flow meter apparatus described above, found no statistically significant difference between MTA and AH Plus [39].

Sönmez et al. (2012) reported that the sealing abilities of AH Plus and MTA were similar, while MTA Fillapex showed more micro-infiltrations than the other two materials [40].

The data on sealing ability are not clearly in favor of epoxy resin-based cements, especially if the study models that have foreseen the measurement by fluid filtration are taken into consideration, while for the bacterial models of microleakage, there is more agreement than the data above when the model foresees a measurement time of at least 90 days.

One limitation of this systematic review is the intermediate level (risk of bias) of the studies included in the review. The intermediate value was mainly caused by the unclear text of the articles in the allocation sequence, randomization, and blinding (risk of bias, Table 4 column 5) and gives a number of samples that are not always adequate to support a robust statistical analysis (risk of bias, Table 4 column 2).

## 5 Conclusion

We can conclude that, even with the limitations of this study, the sealing abilities of epoxy resin-based cements, based on a bacterial micro-infiltration model, are higher than those of tricalcium silicate-based cements for observation periods longer than 90 days.

## Declarations

### Author contribution statement

All authors listed have significantly contributed to the development and the writing of this article.

### Funding statement

This research did not receive any specific grant from funding agencies in the public, commercial, or not-for-profit sectors.

### Data availability statement

No data was used for the research described in the article.

### Declaration of interests statement

The authors declare no conflict of interest.

### Additional information

No additional information is available for this paper.
